# Recent advances in CAR-T cells therapy for colorectal cancer

**DOI:** 10.3389/fimmu.2022.904137

**Published:** 2022-09-27

**Authors:** Xiaoling Qin, Fengjiao Wu, Chang Chen, Qi Li

**Affiliations:** ^1^ Biotherapy Center, Harbin Medical University Cancer Hospital, Harbin, China; ^2^ Department of Pharmacology (State-Province Key Laboratories of Biomedicine-Pharmaceutics of China, Key Laboratory of Cardiovascular Research, Ministry of Education), College of Pharmacy, Department of Pharmacology, Harbin Medical University, Harbin, China

**Keywords:** colorectal cancer, CAR-T cells, antigen, immunotherapy, cell therapy

## Abstract

Colorectal cancer (CRC) is the third most common cancer, with a high mortality rate and a serious impact on people’s life and health. In recent years, adoptive chimeric antigen receptor T (CAR-T) cells therapy has shown well efficacy in the treatment of hematological malignancies, but there are still many problems and challenges in solid tumors such as CRC. For example, the tumor immunosuppressive microenvironment, the low targeting of CAR-T cells, the short time of CAR-T cells *in vivo*, and the limited proliferation capacity of CAR-T cells, CAR-T cells can not effectively infiltrate into the tumor and so on. New approaches have been proposed to address these challenges in CRC, and this review provides a comprehensive overview of the current state of CAR-T cells therapy in CRC.

## Introduction

CRC occurs worldwide, has a high mortality rate and is the third most common cancer (1, 2), which seriously affects human life and health. Due to the rarity of early diagnosis of CRC, existing treatment methods including surgery, chemotherapy and radiotherapy cannot completely inhibit the progression, metastasis and recurrence of CRC when cancer cells infiltrate or metastasize to surrounding tissues (3).

CAR-T cells have shown significant efficacy in immunotargeted therapy of hematologic tumors (4). The United States Food and Drug Administration has approved CAR-T cells for the treatment of hematologic tumors (5). In recent years, basic and clinical studies on CAR-T cells therapy for CRC have been published, and some studies have made encouraging progress (6). However, CAR-T cells face many challenges in the treatment of CRC, limiting their clinical application (7). This article reviews the progress of CAR-T cells therapy for CRC.

**Figure 1:**
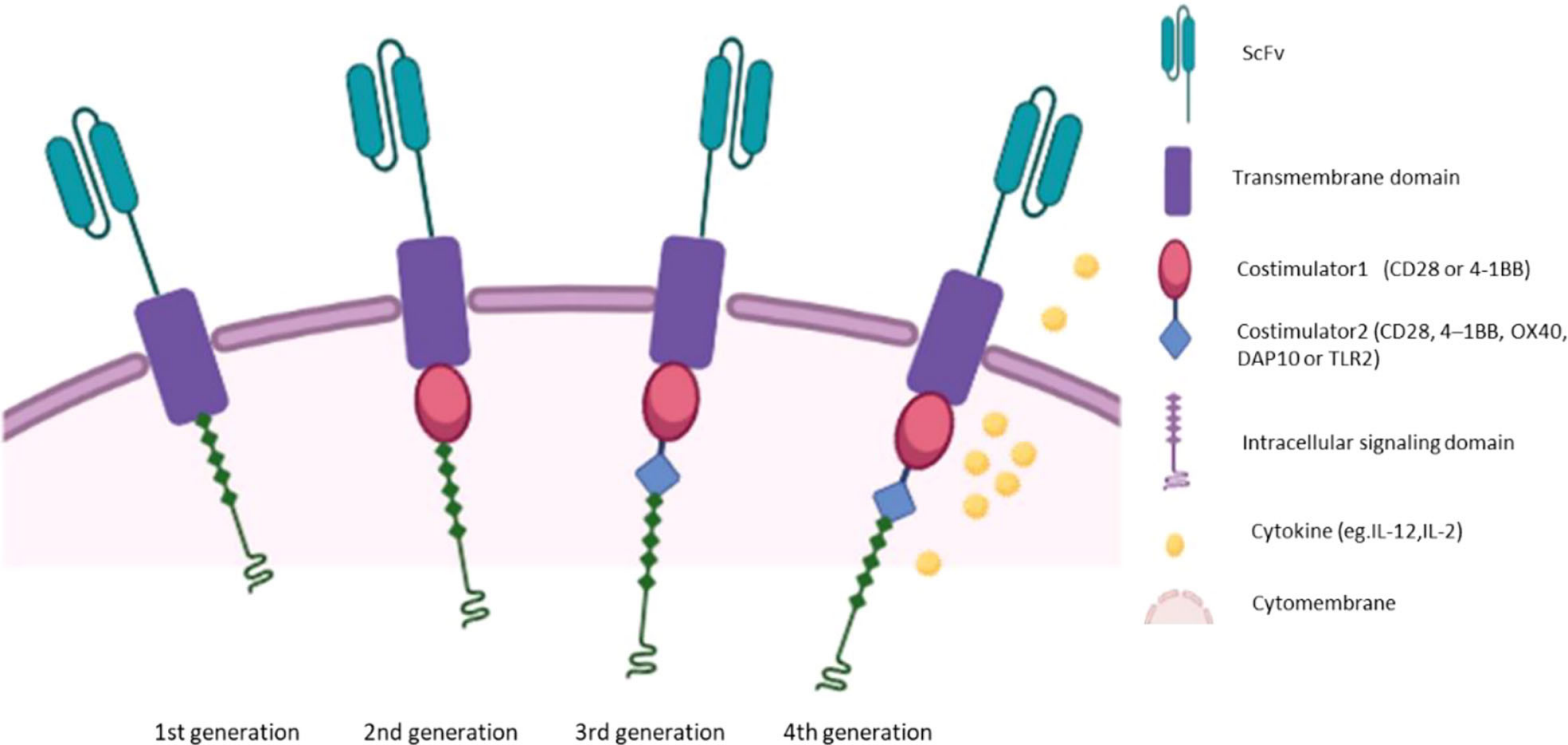
The chimeric antigen receptor (CAR) structure of four generations.

Extracellular region, hinge region, transmembrane region and intracellular signal region are the four components of CAR, and each plays an important role (8). The extracellular domain is usually Fab or single chain variable fragment (scFv) of monoclonal antibody, which has flexible splicing function and determines antigen specificity (9). The hinge domain consists of (Cluster of differentiation4)CD4、CD8、CD28 or IgG4, which connects the extracellular domain to the transmembrane domain (10). The transmembrane domain consists of CD8α, CD4, CD3 ζ, CD28 or ICOS, linking the extracellular domain to the intracellular domain and acting as an anchor of the cell membrane (11, 12). Intracellular signaling domains transmit stimuli into the cell (13). First-generation CAR, which consist of scFv and intracellular CD3ζ molecular signaling domain (14–16), have limited antitumor activity due to the lack of co-stimulation and interleukin signaling (17). The costimulatory domain of the second generation CAR consists of 4-1BB (CD137) or CD28, which mimics costimulatory signals during activation (18). The third generation of CAR has two costimulatory domains, further enhancing the function of CAR (19). The fourth generation CAR is based on the second generation CAR and secretes cytokines such as interleukin2(IL-2) and IL-12 (20, 21). A schematic diagram of the different generations of CARs is shown in **Figure 1**. Recently, researchers designed a combination of focused ultrasound (FUS) and CAR-T cells expressing heat-inducible genes (22). FUS activates heat-inducible genes by controlling local temperature *in vivo (* 22). In animal experiments, CAR-T cells was injected into tumors in mice, and a small ultrasonic transducer was placed on the top of the skin of the tumor area (22). The tumor area was heated through the ultrasonic transducer in the FUS guided by magnetic resonance imaging. Only tumors exposed to ultrasound will be attacked by CAR-T, improving CAR-T targeting (22). This design is expected to be a promising CAR-T.

## Basic experimental of CAR-T cells therapy for CRC

The genetic modification of peripherally derived T lymphocytes with CARs has achieved a remarkable effect in the treatment of hematologic malignancies (23, 24). CAR-T cells therapy for solid tumors still faces many challenges. Recently, there are some advances in CAR-T cells therapy for CRC. The targets of CAR-T cells therapy for CRC include carcino-embryonic antigen(CEA), Mesothelin (MSLN),Guanylyl cyclase C (GUCY2C), epithelial cell adhesion molecule(EpCAM), Human epidermal growth factor receptor-2 (HER2)、Doublecortin-like kinase 1 (DCLK1).

CEA is a glycoprotein formed by cells in the large intestine and a glycoprotein carcinoembryonic antigen, which has been considered as a sensitive marker of CRC (25). At present, there are many basic studies on CAR-T for CEA (26). CAR-T cells have excellent anti-tumor ability when dual targeting CEA and other targets such as CD30 antibody (27).The combination of CEA-CAR-T cells and recombinant human IL‐12(rhIL-12) significantly inhibited the growth of tumor xenografts (28).

MSLN is a cells surface glycoprotein, which is physiologically expressed in peritoneal, pleural and pericardial mesenchymal cells (29). Overexpression of MSLN can be detected in CRC (30). MSLN is an important CAR-T cells target in solid tumors (31, 32). In a recent study, the efficacy of MSLN-CAR-T cells on colon cancer xenografts was investigated. Compared with the control group, the mice in the MSLN-CAR-T cells group had more T lymphocytes in the peripheral blood and more granzyme B infiltrates in the tumor tissue (33). The experimental results showed that the MSLN-CAR-T cells group had a more significant anti-tumor effect (33).

GUCY2C is a binding receptor present in the enterocytes membranes that sustains balance by activating its hormone ligand guanosine or uridine to produce the second messenger cGMP (34). When GUCY2C signaling is blocked, it may lead to the pathogenesis of CRC. However, GUCY2C is expressed in both human primary and metastatic CRC, and GUCY2C is considered to be a tumor marker (35). GUCY2C is highly expressed in 95% of CRC metastasis (36). CAR-T cells targeting hGUCY2C mediated killing of CRC cells expressing hGUCY2C, and were nontoxic to intestinal epithelial cells expressing normal GUCY2C. Such CAR-T cells induce antigen-dependent T-cells activation and cytokine production, thereby enhancing antitumor efficacy (37).

EpCAM is one of the main surface tumor-associated antigens of CRC (38), which can promote the migration, proliferation and tumor growth of colon cancer cells (39). In the experimental treatment of CRC with EpCAM-CAR-T cells, compared with control T cells, EpCAM-CAR-T cells have greater lethality and specificity against cancer cells which express EpCAM (40).

HER2 is overexpressed in CRC (41), and is an important target for CAR-T cells therapy. HER2-CAR-T cells showed strong and particular cytotoxic capacity against colon cancer cells. In mouse models, HER2-CAR-T cells-treated mice showed significant tumor control, significantly improved overall survival, and suppressed distant metastasis of CRC to liver and lung (42).

DCLK1 is an enzyme that regulates epithelial mesenchymal transition (43). Mesenchymal DCLK1 labeling of tumor stem cells in a genetic mouse model of CRC (44). DCLK1-targeted CAR-T cells therapy inhibited xenograft tumor growth in mice without apparent toxicity (45).

Cbl-b is an E3 ubiquitin ligase that mediates ubiquitination, and removal of Cbl-b from CAR-T cells enhances the antitumor activity of CAR-T cells (46). Compared with the control group, Cbl-b ^-/-^CAR-T cells significantly enhanced the killing ability of CAR-T cells against CRC cells, which was manifested by increased secretion of IFN-γ, TNF-α and granzyme B (47).

## Challenges

Adoptive T cells therapy is a new option for tumor patients, but its efficacy is affected by various factors, it is imperative to find relevant strategies to solve the problem.

### Immunosuppression in the tumor microenvironment

Hypoxia, acidic microenvironment and lack of substances necessary for the survival, proliferation and activation of T lymphocytes in tumor tissues will lead to immunosuppressive microenvironment, thereby weakening the killing effect of CAR-T cells on tumor cells (7). Tumor immunosuppressive microenvironment includes suppressive immune cells such as regulatory T cells (Treg), myeloid-derived suppressor cells (MDSC), immunosuppressive cytokines such as IL-10 and TGF-β, immunosuppressive molecules such as PD-1, and other molecules such as indoleamine dioxygenase 2-3 (IDO1) (7, 48). The immunosuppressive microenvironment promotes tumor immune escape (49). The occurrence and development of tumor are correlated strongly with immune escape (50), in which immune checkpoints play an important role (51). Programmed cell death protein 1 (PD-1) and its ligand PD-L1 are significant immune checkpoint proteins (52). PD-1 is an immune checkpoint receptor expressed in T lymphocytes, and PD-L1 is expressed mainly in the tumor microenvironment (53). When PD-1 encounters antigens, its expression is increased and binds to its ligand PD-L1, thereby inhibiting the immune response function of T cells and mediating immune suppression (54).

### CAR-T cells does not effectively chemotaxis to tumor tissue

One of the challenges of CAR-T cells therapy for solid malignancies is the specific recognition of targeted antigens (55). Currently, the majority of tumor target antigens recognized by CAR-T cells are also expressed in normal cells, so when CAR-T cells are used to treat tumors, the therapeutic effect is ineffective (7). Meanwhile, CAR-T cells can also injury normal tissues and cause toxicity *in vitro (* 7).

### CAR-T cells can not proliferate and persist in the blood or tumor area

The persistence and proliferation of CAR-T cells in blood or tumor are important factors for the efficacy of CAR-T cells in cancer treatment (56). Firstly, different costimulatory molecules of CAR affect the survival and proliferation of CAR-T cells (57). Secondly, in the tumor microenvironment, there are a series of factors that affect the survival, proliferation and induce the failure of CAR-T cells. For example, when CAR-T cells are in chronic T helper 2 cells(Th2) inflammation state, their expansion ability is weakened and the number of apoptotic cells is increased (58). Thirdly, TGF-β and adenosine significantly inhibit the tumor cytotoxicity of CD8 + T cells by inhibiting the expression of granzyme (59, 60). In addition, the hypoxic acid microenvironment in the local tumor can cause damage to CAR-T cells, in which lactic acid accumulation can inhibit the production of IL-2, thereby affecting the proliferation and function of CAR-T cells (61). Further, the PD-1/PD-L1 axis affects the survival and function of CAR-T cells (62). Transcription factors T-bet and B lymphocyte-induced maturation protein 1 (Blimp1) regulate early CD8+T lymphocytes (63, 64). Forkhead box protein O1 (FoxO1) can regulate memory CD8+ T cell differentiation (65).

### The level of CAR-T cells invasion in tumor tissue was low

When a CAR-T cells is used to treat a tumor, the CAR-T cells must reach the site of the tumor to perform their tumor-killing function (7). In solid tumors, CAR-T cells must overcome multiple obstacles to reach the tumor site, such as blood vessels and the tumor’s stroma (66, 67). Primarily, when intravenous infusion of CAR-T cells in the treatment of CRC, CAR-T cells must cross the vascular barrier and interstitial barrier to enter the tumor site to exert its efficacy (66). Intratumoral vascular beds and interstitial abnormalities are the key factors affecting the efficacy (66). Then, the inability of many T cells to reach the cancer cells may depend on the lack of surface-expressed chemokine receptor that match chemokine expressed in the tumor or tumor stroma (68). When the chemokines/chemokine receptors axis is mismatched, tumor cells secrete trace amounts of chemokines, resulting in the inability of T cells to reach the tumor tissues (68). For example, CXCL10 can make a variety of antitumor lymphocytes chemotactic to tumor tissues, such as CD8+ T cells, and is associated with T-lymphocytes infiltration in solid tumors (69).

## Strategies

### Develop drugs and measures that can improve the tumor microenvironment

In order to improve the tumor microenvironment to improve the anti-tumor efficacy and durability of CAR-T cells, there are currently the following methods.

It is essential that CAR-T cells secrete pro-inflammatory cytokines to protect them from the inhibitory tumor microenvironment. Studies have shown that secreted cytokines such as IL-7 and IL-12 CAR T cells can improve the immunosuppressive microenvironment (70, 71). Mesenchymal Stem Cells (MSCs) are the main components of tumor stroma and have the ability to actively migrate into tumor tissues (72, 73).By making MSCs capable of releasing IL-7 and IL-12 and combining CAR-T cells, researchers found that CAR-T cells could prolong the time of T cells attack on tumors and improve the tumor immunosuppressive microenvironment (74). IDO1 degrades tryptophan, an essential amino acid for T cells, which is required for T cells survival and immune responses (75). The expression of IDO1 is inhibited by miR-153 (76). When miR-153 was overexpressed in tumor cells, the tumor immunosuppressive environment was improved, CAR-T cells targeting epidermal growth factor receptor variant III(EGFRIII) were more effective in killing colon cancer cells overexpressing miR-153 (77). CD30 signaling can promote the differentiation of T cells to Th2, which has immunosuppressive function (78). In CRC, CAR-T cells dual targeting CD30 and CEA can produce a more significant proinflammatory response, manifested by higher granzyme B and perforin levels In T cells, which improves the ability of CAR-T cells to attack the tumor (27). IL-10 binds to its cognate receptor IL-10R to cause a wide range of immunosuppressive functions (79, 80). Recent studies have shown that CAR-T cells combined with IL-10 monoclonal antibody (mAb) can partially alleviate bone marrow cell-mediated immunosuppression by blocking IL-10 signaling, while promoting CAR-T cells expansion and enhancing killing effect, thereby increasing anti-tumor function (81).

Guo and his team demonstrated that intravenous injection of live attenuated Brucella in mice can promote the tumor microenvironment to a proinflammatory state, enhance the anti-tumor immunity of T cells, and reduce the resistance of tumors to CAR-T cells (82). Dopamine treatment can promote the differentiation of CD8+ T lymphocytes into CD103+ tissue-resident memory CD8+ T lymphocytes (TRM), and TRM can trigger stronger anti-tumor immunity. Moreover, dopamine treatment enhanced the anti-tumor function of CAR-T cells (83).

In addition, blockade of immune checkpoints can improve immunosuppression. Adding genes expressing PD-1 negative receptors to CAR-T cells can block intracellular immune checkpoints and enhance the lethality to target cells (84, 85). Investigators also used clustered regularly interspaced short palindromic repeats (CRISPR) and CRISPR-associated protein 9 (Cas9) (CRISPR-Cas9) systems to knock down the expression of PD-1 in CAR-T cells and achieved excellent preclinical efficacy by blocking PD-1/PD-L1 induced suppression of T-cell immune function (86–89).

### Improve CAR-T cells targeting of tumors

Targeting multiple antigens and application of novel CAR can improve the targeting of CAR-T cells. Jiang and colleagues constructed a dual CAR system containing two extracellular domains, NKG2D and PD-1, and showed that such CAR-T cells effectively eliminated target cancer cells (90). CAR-T cells that dual target CD30 and CEA have a more specific ability to kill tumor cells, which is manifested by blocking the inhibition of cytotoxic T lymphocyte immune function induced by CD30 (27). In addition, when using the novel inhibitory CAR (iCAR) construct, the iCAR can trigger inhibitory signals when CAR-T cells are present in normal tissues, thereby inhibiting T cell function, avoiding the attack of normal tissues, and enhancing the targeting of tumor tissues (7). Additionally, switchable CAR T cells can increase their targeting, with the “switch” acting as a bridge between tumor cells and T cells, allowing T cells to specifically kill tumor cells (91). Besides, the combination of focused ultrasound (FUS) and CAR-T cells, in which only tumors exposed to ultrasound are attacked by CAR-T cells, also improves CAR-T cells targeting (22).

### Amplification and long-term presence of CAR-T cells

How to maintain sustained expansion of CAR-T cells *in vivo* is a common consideration in the treatment of solid tumors with CAR-T cells. Cytokines such as IL-2, IL-7, IL-12 and IL-15 play an important role in T cells activation, proliferation and immune response (92–94). However, the content of immune stimulatory cytokines in the tumor microenvironment is very low. There are now several therapies for combining cytokines with CAR-T cells to treat tumors. CEA-CAR-T cells combined with rhIL-12 can increase the multiplication, persistence and cytokines release of CEA-CAR-T cells *in vivo (* 28). When MSCs that can release IL-7 and IL-12 are used in combination with CAR-T cells, CAR-T cells survive longer and have better expansion ability *in vivo*, thereby improving the anti-tumor response (74). Li and his team demonstrated that inhibition of Wnt significantly inhibited TGF-β expression in tumor tissues and improved T cells infiltration (95). Moreover, after the inhibition of Wnt, the contents of T-bet and FoxO1 in the nucleus of CAR-T cells increased, and the expression of BLIMP1 increased, indicating that the inhibition of Wnt can make CAR-T cells early kill tumor function and differentiate into memory T lymphocytes (95). CD133 is expressed in cancer cells of various epithelial cell origins (96). A phase I trial of CAR-T cells targeting CD133 (CAR-T-133) in the treatment of advanced metastatic malignancies has found that CAR-T-133 cells can persist *in vivo* through multiple infusions and increase the content of immunostimulatory cytokines, which makes valid disease clearance and prevention of relapse possible (97). Previous studies have shown that increasing telomerase activity in CAR-T cells can enhance their proliferation ability and delay senescence (98). Other studies have shown that the costimulatory domain 4-1BB of CAR-T cells can improve the exhaustion of T cells and enhance their persistence *in vivo* (99).

### Increased CAR-T cells invasion in tumors

Targeting tumor blood vessels and stroma and increasing the expression of chemokines are important methods to improve CAR-T cells infiltration into tumor tissues (100). Vascular blocker combretastatin A4 phosphate (CA4P) is a vascular interfering agent with high selectivity for tumor vascular system (101). Targeting CA4P can block the VE-cadherin signaling pathway, affect the stability of microtubule polymerization of tumor cell-related vascular endothelial cells, induce cell apoptosis, destroy the vascular system, reduce the blood supply in the tumor, and lead to tumor cell necrosis in the tumor tissue (101). CA4P combined with HER2-CAR-T cells therapy has a better antitumor effect than CA4P or HER2-CAR-T cell therapy alone, which can destroy tumor blood vessels, thereby promoting the infiltration of T cells into tumor tissues and enhancing the proliferation of CAR T cells (102). Vascular endothelial growth factor (VEGF)/VEGFR axis can promote the generation of vascular endothelial cells, which is a key signaling pathway of angiogenesis (103). VEGFR -targeting CAR T cells can disrupt vascular structures and obviously inhibit xenograft tumor growth, invasion, and metastasis (104). Cancer-associated fibroblasts (CAFs) are important components of tumor stroma (105). Fibroblast activating protein (FAP) is over expression in CAFs and suppresses tumor immune response by promoting the recruitment of immunosuppressive cells (106). At present, FAP-targeted CAR-T cells have achieved certain preclinical and clinical efficacy in solid tumors (107, 108). When the Wnt signaling pathway is blocked, it can up-regulate the expression of chemokine CXCL10, improve T cells tumor infiltration in cancer models, and improve the efficacy of CAR-T cells in CRC treatment (95, 109).

## Clinical trials

In the past few years, immune cell therapy has been increasingly used in multicentre clinical trials. Multiple clinical trials targeting tumor antigens have been approved, including CEA, MSLN, EpCAM, HER2 and antigens, as well as NK group 2 member D ligands (NKG2DL), Mucin-1 (MUC1), B7-H3 (CD276), CD133, mesenchymal epithelial transfer factor(c-Met), which is overexpressed in colorectal cancer, can be used as a target for CAR-T cells. In **Table 1**, we summarized the clinical information available on ClinicalTrials.gov regarding CAR-T-cells therapy for CRC.

**Table 1:** Clinical trial of CAR-T cells in CRC(https://clinicaltrials.gov/).

Antigen	phase	Clinicaltrials. gov identifier	CAR-T Cells Treatment		Recruitment Status	
NKG2DL	Early Phase 1	NCT05248048	NA		Recruiting	
	Phase 1	NCT04550663	NA		Not yet recruiting	
	Phase 1	NCT03370198	3 DL: 3 × 10^8^–3 × 10^9^ cells/d(3ds)		Active, not recruiting	
	Phase 1	NCT04107142	3DL:3 x 10^8^- 3 x 10^9^ CAR-γδ T cells/d(4ds)		Unknown	
	Phase 1	NCT03310008	3 DL: 10^8^–10^9^cells/d (3 ds) and FOLFOX		Active, not recruiting	
	Phase 1	NCT03692429	3 DL:1-100x10^8^cells/d (3 ds) andFOLFOX		Recruiting	
CEA	Phase 1	NCT02850536	1 × 10^10^ cells/d(3 ds) with IL2		Completed	
	Phase 1	NCT02416466	1 × 10^10^ cells/d(3ds) with IL-2		Completed
	Early Phase 1	NCT04513431	NA		Not yet recruiting
	Phase 1	NCT05240950	3DL:1- 6×10^6^/kg anti-CEA CAR-T (+) cells(1d)		Recruiting
	Phase 1 Phase 2	NCT04348643	NA		Recruiting
	Phase 1	NCT02349724	5 DL: 10^5^–10^8^ CAR+ cells/kg (split: 10%,30% and 60% per day)		Completed
	Phase 1	NCT03682744	NA		Active, not recruiting
	Phase 1 Phase 2	NCT02959151	1.25~4×10^7^ CAR+T cells/cm3 tumor bulk(1d)		Unknown
MSLN	Phase 1	NCT05089266	NA		Not yet recruiting	
	Early Phase 1	NCT04503980	4DL:1×10^5^-3×10^6^αPD1 MSLN-CAR+ T cells/kg(1d)		Recruiting
EpCAM	Phase 1	NCT05028933	3DL:3-10×10^5^EPCAM CAR-T/kg(1d)		Recruiting	
MUC1	Phase 1	NCT05239143	NA		Recruiting	
	Phase 1 Phase 2	NCT02617134	NA		Unknown	
HER2	Phase 1	NCT03740256	7 DL: 1–100 × 10^6^Cells(1d) and oncolytic adenovirus CAdVEC intra-tumor injection		Recruiting	
B7-H3	Phase 1	NCT05190185	3DL:1-100×10^6^ CAR-T/kg(1d)		Recruiting	
EGFR	Phase 1	NCT03542799	NA		Unknown	
	Phase 1 Phase 2	NCT03152435	NA		Unknown	
CD133	Phase 1 Phase 2	NCT02541370	0.5–2 × 10^6^ cells/kg(2ds)		Completed	
c-Met	Phase 1 Phase 2	NCT03638206	NA		Recruiting	

NA, not available; d(s), dose(s); DL, dose levels.

In a phase I trial of CEA + CRC patients treated with CEA-CAR-T cells (NCT02349724), five dose-escalation CAR-T cells were administered to 10 patients with relapsed and refractory CRC metastases. No serious adverse events related to CAR-T cells therapy were observed in the trial (6). Among the 10 patients, 7 were stable after CAR-T cells therapy, of which 2 were stable for more than 30 weeks and 2 showed tumor shrinkage (6).

A phase 1B hepatic Immunotherapy for Metastases-selective internal irradiation therapy (HITM-SIR) trial was conducted in patients with liver metastases from CRC (NCT01373047). Six of them received anti-CEA CAR-T hepatic artery infusions (HAIs) and SIRT. Significant reductions in Granulocyte macrophage colony stimulating factor (GM-CSF), GM-CSF-R, IDO, and Programmed death ligand-1(PD-L1) were observed after HITM CAR-T HAI treatment, suggesting a reversal of immunosuppressed hepatic tumor microenvironment (TME). Subsequent increases in IL-2 and IL-6 in tumor biopsies after infusion further demonstrated pro-inflammatory liver TME. The median survival of patients in the trial was 8 months (110).

## Conclusions

There are many approaches to CRC adoptive cell therapy, of which CAR-T cells are one of the most researched and promising, although clinical studies are still in the early stages of clinical trials. Many studies have demonstrated the efficacy and safety of CAR-T cells in the treatment of CRC. However, the therapy faces many challenges that limit its clinical application. In addition, CAR-T cells therapy can cause a number of toxic effects, the most common of which is cytokine release syndrome (CRS), which is a cytokine secretion response after CAR-T cells infusion (111). CRS has a series of non-characteristic manifestations, such as fever, nausea, decreased cardiac function, and hypotension (112). It can also cause other systemic toxicity, such as dyspnea, respiratory failure, arrhythmia, elevated myocardial enzymes, cardiac insufficiency, liver insufficiency, gastrointestinal reaction, coagulation dysfunction, muscle injury, neurotoxic allergy, etc (112). Only when these problems are effectively addressed can the efficacy of CAR-T cells therapy for CRC be improved and more patients receive effective treatment. In conclusion, CAR-T cells are a promising treatment for CRC and further research is needed.

## Author contributions

CC and QL designed this manuscript. XQ wrote the main manuscript text. FW prepared figure and table. CC and QL revised the article. All authors contributed to the article and approved the submitted version.

## Funding

This work was supported by the National Natural Science Foundation of China [grant number 81800242 and 81970382]; The Science foundation of Haiyan [grant number JJMS2022-16].

## Conflict of interest

The authors declare that the research was conducted in the absence of any commercial or financial relationships that could be construed as a potential conflict of interest.

## Publisher’s note

All claims expressed in this article are solely those of the authors and do not necessarily represent those of their affiliated organizations, or those of the publisher, the editors and the reviewers. Any product that may be evaluated in this article, or claim that may be made by its manufacturer, is not guaranteed or endorsed by the publisher.

## References

[1] Schrock AB Ouyang C Sandhu J Sokol E Jin D Ross JS. Tumor mutational burden is predictive of response to immune checkpoint inhibitors in MSI-high metastatic colorectal cancer. Ann Oncol (2019) 30(7):1096–103. doi: 10.1093/annonc/mdz134 31038663

[2] Sung H Ferlay J Siegel RL Laversanne M Soerjomataram I Jemal A. Global cancer statistics 2020: GLOBOCAN estimates of incidence and mortality worldwide for 36 cancers in 185 countries. CA Cancer J Clin (2021) 71(3):209–49. doi: 10.3322/caac.21660 33538338

[3] Sawicki T Ruszkowska M Danielewicz A Niedźwiedzka E Arłukowicz T Przybyłowicz KE. A review of colorectal cancer in terms of epidemiology, risk factors, development, symptoms and diagnosis. Cancers (Basel) (2021) 13(9):2025. doi: 10.3390/cancers13092025 33922197 PMC8122718

[4] June CH O'Connor RS Kawalekar OU Ghassemi S Milone MC. CAR T cell immunotherapy for human cancer. Science (2018) 359(6382):1361–5. doi: 10.1126/science.aar6711 29567707

[5] Fournier C Martin F Zitvogel L Kroemer G Galluzzi L Apetoh L. Trial watch: Adoptively transferred cells for anticancer immunotherapy. Oncoimmunology (2017) 6(11):e1363139. doi: 10.1080/2162402X.2017.1363139 29147628 PMC5674950

[6] Zhang C Wang Z Yang Z Wang M Li S Li Y. Phase I escalating-dose trial of CAR-T therapy targeting CEA(+) metastatic colorectal cancers. Mol Ther (2017) 25(5):1248–58. doi: 10.1016/j.ymthe.2017.03.010 PMC5417843 28366766

[7] D’Aloia MM Zizzari IG Sacchetti B Pierelli L Alimandi M. CAR-T cells: The long and winding road to solid tumors. Cell Death Dis (2018) 9(3):282. doi: 10.1038/s41419-018-0278-6 29449531 PMC5833816

[8] Lipowska-Bhalla G Gilham DE Hawkins RE Rothwell DG. Targeted immunotherapy of cancer with CAR T cells: Achievements and challenges. Cancer Immunol Immunother (2012) 61(7):953–62. doi: 10.1007/s00262-012-1254-0 PMC11028843 22527245

[9] Zhang G Wang L Cui H Wang X Zhang G Ma J. Anti-melanoma activity of T cells redirected with a TCR-like chimeric antigen receptor. Sci Rep (2014) 4:3571. doi: 10.1038/srep03571 24389689 PMC3880964

[10] Hudecek M Sommermeyer D Kosasih PL Silva-Benedict A Liu L Rader C. The nonsignaling extracellular spacer domain of chimeric antigen receptors is decisive for *in vivo* antitumor activity. Cancer Immunol Res (2015) 3(2):125–35. doi: 10.1158/2326-6066.CIR-14-0127 PMC4692801 25212991

[11] Bridgeman JS Hawkins RE Bagley S Blaylock M Holland M Gilham DE. The optimal antigen response of chimeric antigen receptors harboring the CD3zeta transmembrane domain is dependent upon incorporation of the receptor into the endogenous TCR/CD3 complex. J Immunol (2010) 184(12):6938–49. doi: 10.4049/jimmunol.0901766 20483753

[12] Guedan S Posey AD Jr Shaw C Wing A Da T Patel PR. Enhancing CAR T cell persistence through ICOS and 4-1BB costimulation. JCI Insight (2018) 3(1):e96976. doi: 10.1172/jci.insight.96976 PMC5821198 29321369

[13] Zhang C Liu J Zhong JF Zhang X. Engineering CAR-T cells. biomark Res (2017) 5:22. doi: 10.1186/s40364-017-0102-y 28652918 PMC5482931

[14] Stancovski I Schindler DG Waks T Yarden Y Sela M Eshhar Z. Targeting of T lymphocytes to Neu/HER2-expressing cells using chimeric single chain fv receptors. J Immunol (1993) 151(11):6577–82. 7902379

[15] Hwu P Shafer GE Treisman J Schindler DG Gross G Cowherd R. Lysis of ovarian cancer cells by human lymphocytes redirected with a chimeric gene composed of an antibody variable region and the fc receptor gamma chain. J Exp Med (1993) 178(1):361–6. doi: 10.1084/jem.178.1.361 PMC2191075 8315392

[16] Hwu P Yang JC Cowherd R Treisman J Shafer GE Eshhar Z. *In vivo* antitumor activity of T cells redirected with chimeric antibody/T-cell receptor genes. Cancer Res (1995) 55(15):3369–73. 7614473

[17] Thistlethwaite FC Gilham DE Guest RD Rothwell DG Pillai M Burt DJ. The clinical efficacy of first-generation carcinoembryonic antigen (CEACAM5)-specific CAR T cells is limited by poor persistence and transient pre-conditioning-dependent respiratory toxicity. Cancer Immunol Immunother (2017) 66(11):1425–36. doi: 10.1007/s00262-017-2034-7 PMC5645435 28660319

[18] Subklewe M von Bergwelt-Baildon M Humpe A. Chimeric antigen receptor T cells: A race to revolutionize cancer therapy. Transfus. Med Hemother. (2019) 46(1):15–24. doi: 10.1159/000496870 31244578 PMC6558337

[19] Huang R Li X He Y Zhu W Gao L Liu Y. Recent advances in CAR-T cell engineering. J Hematol Oncol (2020) 13(1):86. doi: 10.1186/s13045-020-00910-5 32616000 PMC7333410

[20] Tokarew N Ogonek J Endres S von Bergwelt-Baildon M Kobold S. Teaching an old dog new tricks: next-generation CAR T cells. Br J Cancer (2019) 120(1):26–37. doi: 10.1038/s41416-018-0325-1 30413825 PMC6325111

[21] Yeku OO Brentjens RJ. Armored CAR T-cells: utilizing cytokines and pro-inflammatory ligands to enhance CAR T-cell anti-tumour efficacy. Biochem Soc Trans (2016) 44(2):412–8. doi: 10.1042/BST20150291 PMC5529098 27068948

[22] Wu Y Liu Y Huang Z Wang X Jin Z Li J. Control of the activity of CAR-T cells within tumours *via* focused ultrasound. Nat BioMed Eng (2021) 5(11):1336–47. doi: 10.1038/s41551-021-00779-w PMC9015817 34385696

[23] Porter DL Levine BL Kalos M Bagg A June CH. Chimeric antigen receptor-modified T cells in chronic lymphoid leukemia. N Engl J Med (2011) 365(8):725–33. doi: 10.1056/NEJMoa1103849 PMC3387277 21830940

[24] Maude SL Frey N Shaw PA Aplenc R Barrett DM Bunin NJ. Chimeric antigen receptor T cells for sustained remissions in leukemia. N Engl J Med (2014) 371(16):1507–17. doi: 10.1056/NEJMoa1407222 PMC4267531 25317870

[25] Jelski W Mroczko B. Biochemical markers of colorectal cancer - present and future. Cancer Manag. Res (2020) 12:4789–97. doi: 10.2147/CMAR.S253369 PMC7319530 32606968

[26] Fan J Das JK Xiong X Chen H Song J. Development of CAR-T cell persistence in adoptive immunotherapy of solid tumors. Front Oncol (2020) 10:574860. doi: 10.3389/fonc.2020.574860 33489881 PMC7815927

[27] Hombach AA Rappl G Abken H. Blocking CD30 on T cells by a dual specific CAR for CD30 and colon cancer antigens improves the CAR T cell response against CD30(-) tumors. Mol Ther (2019) 27(10):1825–35. doi: 10.1016/j.ymthe.2019.06.007 PMC6822283 31331813

[28] Chi X Yang P Zhang E Gu J Xu H Li M. Significantly increased anti-tumor activity of carcinoembryonic antigen-specific chimeric antigen receptor T cells in combination with recombinant human IL-12. Cancer Med (2019) 8(10):4753–65. doi: 10.1002/cam4.2361 PMC6712469 31237116

[29] Hassan R Thomas A Alewine C Le DT Jaffee EM Pastan I. Mesothelin immunotherapy for cancer: Ready for prime time? J Clin Oncol (2016) 34(34):4171–9. doi: 10.1200/JCO.2016.68.3672 PMC5477819 27863199

[30] Morello A Sadelain M Adusumilli PS. Mesothelin-targeted CARs: Driving T cells to solid tumors. Cancer Discovery (2016) 6(2):133–46. doi: 10.1158/2159-8290.CD-15-0583 PMC4744527 26503962

[31] Liang Z Dong J Yang N Li SD Yang ZY Huang R. Tandem CAR-T cells targeting FOLR1 and MSLN enhance the antitumor effects in ovarian cancer. Int J Biol Sci (2021) 17(15):4365–76. doi: 10.7150/ijbs.63181 PMC8579462 34803504

[32] Schoutrop E Renken S Micallef Nilsson I Hahn P Poiret T Kiessling R. Trogocytosis and fratricide killing impede MSLN-directed CAR T cell functionality. Oncoimmunology (2022) 11(1):2093426. doi: 10.1080/2162402X.2022.2093426 35898704 PMC9313125

[33] Zhang Q Liu G Liu J Yang M Fu J Liu G. The antitumor capacity of mesothelin-CAR-T cells in targeting solid tumors in mice. Mol Ther Oncolytics (2021) 20:556–68. doi: 10.1016/j.omto.2021.02.013 PMC7943972 33738341

[34] Yarla NS Gali H Pathuri G Smriti S Farooqui M Panneerselvam J. Targeting the paracrine hormone-dependent guanylate cyclase/cGMP/phosphodiesterases signaling pathway for colorectal cancer prevention. Semin Cancer Biol (2019) 56:168–74. doi: 10.1016/j.semcancer.2018.08.011 30189250

[35] Entezari AA Snook AE Waldman SA. Guanylyl cyclase 2C (GUCY2C) in gastrointestinal cancers: recent innovations and therapeutic potential. Expert Opin Ther Targets (2021) 25(5):335–46. doi: 10.1080/14728222.2021.1937124 PMC8363580 34056991

[36] Birbe R Palazzo JP Walters R Weinberg D Schulz S Waldman SA. Guanylyl cyclase c is a marker of intestinal metaplasia, dysplasia, and adenocarcinoma of the gastrointestinal tract. Hum Pathol (2005) 36(2):170–9. doi: 10.1016/j.humpath.2004.12.002 15754294

[37] Magee MS Abraham TS Baybutt TR Flickinger JC Jr Ridge NA Marszalowicz GP. Human GUCY2C-targeted chimeric antigen receptor (CAR)-expressing T cells eliminate colorectal cancer metastases. Cancer Immunol Res (2018) 6(5):509–16. doi: 10.1158/2326-6066.CIR-16-0362 PMC5932207 29615399

[38] Herlyn D Herlyn M Steplewski Z Koprowski H. Monoclonal antibodies in cell-mediated cytotoxicity against human melanoma and colorectal carcinoma. Eur J Immunol (1979) 9(8):657–9. doi: 10.1002/eji.1830090817 499332

[39] Liang KH Tso HC Hung SH Kuan II Lai JK Ke FY. Extracellular domain of EpCAM enhances tumor progression through EGFR signaling in colon cancer cells. Cancer Lett (2018) 433:165–75. doi: 10.1016/j.canlet.2018.06.040 29981429

[40] Feng M Jin JQ Xia L Xiao T Mei S Wang X. Pharmacological inhibition of β-catenin/BCL9 interaction overcomes resistance to immune checkpoint blockades by modulating t(reg) cells. Sci Adv (2019) 5(5):eaau5240. doi: 10.1126/sciadv.aau5240 31086813 PMC6506245

[41] La Salvia A Lopez-Gomez V Garcia-Carbonero R. HER2-targeted therapy: an emerging strategy in advanced colorectal cancer. Expert Opin Investig Drugs (2019) 28(1):29–38. doi: 10.1080/13543784.2019.1555583 30513002

[42] Xu J Meng Q Sun H Zhang X Yun J Li B. HER2-specific chimeric antigen receptor-T cells for targeted therapy of metastatic colorectal cancer. Cell Death Dis (2021) 12(12):1109. doi: 10.1038/s41419-021-04100-0 34839348 PMC8627513

[43] Sureban SM May R Mondalek FG Qu D Ponnurangam S Pantazis P. Nanoparticle-based delivery of siDCAMKL-1 increases microRNA-144 and inhibits colorectal cancer tumor growth *via* a notch-1 dependent mechanism. J Nanobiotechno. (2011) 9:40. doi: 10.1186/1477-3155-9-40 PMC3200989 21929751

[44] Kim JH Park SY Jeon SE Choi JH Lee CJ Jang TY. DCLK1 promotes colorectal cancer stemness and aggressiveness *via* the XRCC5/COX2 axis. Theranostics (2022) 12(12):5258–71. doi: 10.7150/thno.72037 PMC9330537 35910805

[45] Sureban SM Berahovich R Zhou H Xu S Wu L Ding K. DCLK1 monoclonal antibody-based CAR-T cells as a novel treatment strategy against human colorectal cancers. Cancers (Basel) (2019) 12(1):54. doi: 10.3390/cancers12010054 PMC7016951 31878090

[46] Venuprasad K. Cbl-b and itch: Key regulators of peripheral T-cell tolerance. Cancer Res (2010) 70(8):3009–12. doi: 10.1158/0008-5472.CAN-09-4076 PMC2856114 20395198

[47] Kumar J Kumar R Kumar Singh A Tsakem EL Kathania M Riese MJ. Deletion of cbl-b inhibits CD8(+) T-cell exhaustion and promotes CAR T-cell function. J Immunother Cancer (2021) 9(1):e001688. doi: 10.1136/jitc-2020-001688 33462140 PMC7813298

[48] Klemm F Joyce JA. Microenvironmental regulation of therapeutic response in cancer. Trends Cell Biol (2015) 25(4):198–213. doi: 10.1016/j.tcb.2014.11.006 25540894 PMC5424264

[49] Tang H Qiao J Fu YX. Immunotherapy and tumor microenvironment. Cancer Lett (2016) 370(1):85–90. doi: 10.1016/j.canlet.2015.10.009 26477683 PMC4725050

[50] Li F Zhang R Li S Liu J. IDO1: An important immunotherapy target in cancer treatment. Int Immunopharmacol. (2017) 47:70–7. doi: 10.1016/j.intimp.2017.03.024 28365507

[51] Pardoll DM. The blockade of immune checkpoints in cancer immunotherapy. Nat Rev Cancer (2012) 12(4):252–64. doi: 10.1038/nrc3239 PMC4856023 22437870

[52] Iwai Y Ishida M Tanaka Y Okazaki T Honjo T Minato N. Involvement of PD-L1 on tumor cells in the escape from host immune system and tumor immunotherapy by PD-L1 blockade. Proc Natl Acad Sci U.S.A. (2002) 99(19):12293–7. doi: 10.1073/pnas.192461099 PMC129438 12218188

[53] Okazaki T Honjo T. PD-1 and PD-1 ligands: from discovery to clinical application. Int Immunol (2007) 19(7):813–24. doi: 10.1093/intimm/dxm057 17606980

[54] Jiang X Wang J Deng X Xiong F Ge J Xiang B. Role of the tumor microenvironment in PD-L1/PD-1-mediated tumor immune escape. Mol Cancer (2019) 18(1):10. doi: 10.1186/s12943-018-0928-4 30646912 PMC6332843

[55] Ma S Li X Wang X Cheng L Li Z Zhang C. Current progress in CAR-T cell therapy for solid tumors. Int J Biol Sci (2019) 15(12):2548–60. doi: 10.7150/ijbs.34213 PMC6854376 31754328

[56] Ghorashian S Kramer AM Onuoha S Wright G Bartram J Richardson R. Enhanced CAR T cell expansion and prolonged persistence in pediatric patients with ALL treated with a low-affinity CD19 CAR. Nat Med (2019) 25(9):1408–14. doi: 10.1038/s41591-019-0549-5 31477906

[57] Weinkove R George P Dasyam N McLellan AD. Selecting costimulatory domains for chimeric antigen receptors: functional and clinical considerations. Clin Transl Immunol (2019) 8(5):e1049. doi: 10.1002/cti2.1049 PMC6511336 31110702

[58] Haabeth OA Lorvik KB Hammarström C Donaldson IM Haraldsen G Bogen B. Inflammation driven by tumour-specific Th1 cells protects against b-cell cancer. Nat Commun (2011) 2:240. doi: 10.1038/ncomms1239 21407206 PMC3072106

[59] Tang N Cheng C Zhang X Qiao M Li N Mu W. TGF-β inhibition *via* CRISPR promotes the long-term efficacy of CAR T cells against solid tumors. JCI Insight (2020) 5(4):e133977. doi: 10.1172/jci.insight.133977 PMC7101140 31999649

[60] Li N Tang N Cheng C Hu T Wei X Han W. Improving the anti-solid tumor efficacy of CAR-T cells by inhibiting adenosine signaling pathway. Oncoimmunology (2020) 9(1):1824643. doi: 10.1080/2162402X.2020.1824643 33457103 PMC7781731

[61] Calcinotto A Filipazzi P Grioni M Iero M De Milito A Ricupito A. Modulation of microenvironment acidity reverses anergy in human and murine tumor-infiltrating T lymphocytes. Cancer Res (2012) 72(11):2746–56. doi: 10.1158/0008-5472.CAN-11-1272 22593198

[62] Lotfinejad P Kazemi T Mokhtarzadeh A Shanehbandi D Jadidi Niaragh F Safaei S. PD-1/PD-L1 axis importance and tumor microenvironment immune cells. Life Sci (2020) 259:118297. doi: 10.1016/j.lfs.2020.118297 32822718

[63] Kallies A Xin A Belz GT Nutt SL. Blimp-1 transcription factor is required for the differentiation of effector CD8(+) T cells and memory responses. Immunity (2009) 31(2):283–95. doi: 10.1016/j.immuni.2009.06.021 19664942

[64] Joshi NS Cui W Chandele A Lee HK Urso DR Hagman J. Inflammation directs memory precursor and short-lived effector CD8(+) T cell fates *via* the graded expression of T-bet transcription factor. Immunity (2007) 27(2):281–95. doi: 10.1016/j.immuni.2007.07.010 PMC2034442 17723218

[65] Hess Michelini R Doedens AL Goldrath AW Hedrick SM. Differentiation of CD8 memory T cells depends on Foxo1. J Exp Med (2013) 210(6):1189–200. doi: 10.1084/jem.20130392 PMC3674697 23712431

[66] Vignali D Kallikourdis M. Improving homing in T cell therapy. Cytokine Growth Factor Rev (2017) 36:107–16. doi: 10.1016/j.cytogfr.2017.06.009 28690108

[67] Hanahan D Coussens LM. Accessories to the crime: functions of cells recruited to the tumor microenvironment. Cancer Cell (2012) 21(3):309–22. doi: 10.1016/j.ccr.2012.02.022 22439926

[68] Moon EK Wang LS Bekdache K Lynn RC Lo A Thorne SH. Intra-tumoral delivery of CXCL11 *via* a vaccinia virus, but not by modified T cells, enhances the efficacy of adoptive T cell therapy and vaccines. Oncoimmunology (2018) 7(3):e1395997. doi: 10.1080/2162402X.2017.1395997 29399394 PMC5790399

[69] Romero JM Grünwald B Jang GH Bavi PP Jhaveri A Masoomian M. A four-chemokine signature is associated with a T-cell-Inflamed phenotype in primary and metastatic pancreatic cancer. Clin Cancer Res (2020) 26(8):1997–2010. doi: 10.1158/1078-0432.CCR-19-2803 31964786

[70] Luo H Su J Sun R Sun Y Wang Y Dong Y. Coexpression of IL7 and CCL21 increases efficacy of CAR-T cells in solid tumors without requiring preconditioned lymphodepletion. Clin Cancer Res (2020) 26(20):5494–505. doi: 10.1158/1078-0432.CCR-20-0777 32816947

[71] Kueberuwa G Su J Sun R Sun Y Wang Y Dong Y. CD19 CAR T cells expressing IL-12 eradicate lymphoma in fully lymphoreplete mice through induction of host immunity. Mol Ther Oncolytics (2018) 8:41–51. doi: 10.1016/j.omto.2017.12.003 29367945 PMC5772011

[72] Melzer C Yang Y Hass R. Interaction of MSC with tumor cells. Cell Commun Signal (2016) 14(1):20. doi: 10.1186/s12964-016-0143-0 27608835 PMC5016940

[73] Relation T Yi T Guess AJ La Perle K Otsuru S Hasgur S. Intratumoral delivery of interferonγ-secreting mesenchymal stromal cells repolarizes tumor-associated macrophages and suppresses neuroblastoma proliferation *in vivo* . Stem Cells (2018) 36(6):915–24. doi: 10.1002/stem.2801 29430789

[74] Hombach AA Geumann U Günther C Hermann FG Abken H. IL7-IL12 engineered mesenchymal stem cells (MSCs) improve a CAR T cell attack against colorectal cancer cells. Cells (2020) 9(4):873. doi: 10.3390/cells9040873 PMC7226757 32260097

[75] Brandacher G Perathoner A Ladurner R Schneeberger S Obrist P Winkler C. Prognostic value of indoleamine 2,3-dioxygenase expression in colorectal cancer: effect on tumor-infiltrating T cells. Clin Cancer Res (2006) 12(4):1144–51. doi: 10.1158/1078-0432.CCR-05-1966 16489067

[76] Zhang W Mao S Shi D Zhang J Zhang Z Guo Y. MicroRNA-153 decreases tryptophan catabolism and inhibits angiogenesis in bladder cancer by targeting indoleamine 2,3-dioxygenase 1. Front Oncol (2019) 9:619. doi: 10.3389/fonc.2019.00619 31355138 PMC6636202

[77] Huang Q Xia J Wang L Wang X Ma X Deng Q. miR-153 suppresses IDO1 expression and enhances CAR T cell immunotherapy. J Hematol Oncol (2018) 11(1):58. doi: 10.1186/s13045-018-0600-x 29685162 PMC5914051

[78] Del Prete G De Carli M D'Elios MM Daniel KC Almerigogna F Alderson M. CD30-mediated signaling promotes the development of human T helper type 2-like T cells. J Exp Med (1995) 182(6):1655–61. doi: 10.1084/jem.182.6.1655 PMC2192264 7500010

[79] Saraiva M Vieira P O’Garra A. Biology and therapeutic potential of interleukin-10. J Exp Med (2020) 217(1):e20190418. doi: 10.1084/jem.20190418 31611251 PMC7037253

[80] Ruffell B Chang-Strachan D Chan V Rosenbusch A Ho CM Pryer N. Macrophage IL-10 blocks CD8+ T cell-dependent responses to chemotherapy by suppressing IL-12 expression in intratumoral dendritic cells. Cancer Cell (2014) 26(5):623–37. doi: 10.1016/j.ccell.2014.09.006 PMC4254570 25446896

[81] Sullivan KM Jiang X Guha P Lausted C Carter JA Hsu C. Blockade of interleukin 10 potentiates antitumour immune function in human colorectal cancer liver metastases. Gut (2022) Jun 15:gutjnl-2021-325808. doi: 10.1136/gutjnl-2021-325808 PMC9872249 35705369

[82] Guo F Das JK Kobayashi KS Qin QM T AF Alaniz RC. Live attenuated bacterium limits cancer resistance to CAR-T therapy by remodeling the tumor microenvironment. J Immunother Cancer (2022) 10(1):e003760. doi: 10.1136/jitc-2021-003760 34987022 PMC8734016

[83] Chen Y Yan SM Pu Z Feng J Tan L Li Y. Dopamine signaling promotes tissue-resident memory differentiation of CD8+ T cells and antitumor immunity. Cancer Res (2022) 82(17):3130–42. doi: 10.1158/0008-5472.CAN-21-4084 35802647

[84] Chen N Morello A Tano Z Adusumilli PS. CAR T-cell intrinsic PD-1 checkpoint blockade: A two-in-one approach for solid tumor immunotherapy. Oncoimmunology (2017) 6(2):e1273302. doi: 10.1080/2162402X.2016.1273302 28344886 PMC5353939

[85] Chen C Gu YM Zhang F Zhang ZC Zhang YT He YD. Construction of PD1/CD28 chimeric-switch receptor enhances anti-tumor ability of c-met CAR-T in gastric cancer. Oncoimmunology (2021) 10(1):1901434. doi: 10.1080/2162402X.2021.1901434 33854821 PMC8018404

[86] Hu W Zi Z Jin Y Li G Shao K Cai Q. CRISPR/Cas9-mediated PD-1 disruption enhances human mesothelin-targeted CAR T cell effector functions. Cancer Immunol Immunother (2019) 68(3):365–77. doi: 10.1007/s00262-018-2281-2 PMC11028344 30523370

[87] Nakazawa T Natsume A Nishimura F Morimoto T Matsuda R Nakamura M. Effect of CRISPR/Cas9-mediated PD-1-Disrupted primary human third-generation CAR-T cells targeting EGFRvIII on *in vitro* human glioblastoma cell growth. Cells (2020) 9(4):998. doi: 10.3390/cells9040998 PMC7227242 32316275

[88] Choi BD Yu X Castano AP Darr H Henderson DB Bouffard AA. CRISPR-Cas9 disruption of PD-1 enhances activity of universal EGFRvIII CAR T cells in a preclinical model of human glioblastoma. J Immunother Cancer (2019) 7(1):304. doi: 10.1186/s40425-019-0806-7 31727131 PMC6857271

[89] Ren J Zhao Y. Advancing chimeric antigen receptor T cell therapy with CRISPR/Cas9. Protein Cell (2017) 8(9):634–43. doi: 10.1007/s13238-017-0410-x PMC5563282 28434148

[90] Jiang G Ng YY Tay JCK Du Z Xiao L Wang S. Dual CAR-T cells to treat cancers co-expressing NKG2D and PD1 ligands in xenograft models of peritoneal metastasis. Cancer Immunol Immunother (2022). doi: 10.1007/s00262-022-03247-9 PMC10992227 35809118

[91] Raj D Yang MH Rodgers D Hampton EN Begum J Mustafa A. Switchable CAR-T cells mediate remission in metastatic pancreatic ductal adenocarcinoma. Gut (2019) 68(6):1052–64. doi: 10.1136/gutjnl-2018-316595 PMC6580747 30121627

[92] Hernandez R LaPorte KM Hsiung S Santos Savio A Malek TR. High-dose IL-2/CD25 fusion protein amplifies vaccine-induced CD4(+) and CD8(+) neoantigen-specific T cells to promote antitumor immunity. J Immunother Cancer (2021) 9(9):e002865. doi: 10.1136/jitc-2021-002865 34475132 PMC8413969

[93] Lee JY Nguyen B Mukhopadhyay A Han M Zhang J Gujar R. Amplification of the CXCR3/CXCL9 axis *via* intratumoral electroporation of plasmid CXCL9 synergizes with plasmid IL-12 therapy to elicit robust anti-tumor immunity. Mol Ther Oncolytics (2022) 25:174–88. doi: 10.1016/j.omto.2022.04.005 PMC9092072 35592387

[94] Watkinson F Nayar SK Rani A Sakellariou CA Elhage O Papaevangelou E. IL-15 upregulates telomerase expression and potently increases proliferative capacity of NK, NKT-like, and CD8 T cells. Front Immunol (2020) 11:594620. doi: 10.3389/fimmu.2020.594620 33537030 PMC7848219

[95] Li W Zhou Y Wu Z Shi Y Tian E Zhu Y. Targeting wnt signaling in the tumor immune microenvironment to enhancing EpCAM CAR T-cell therapy. Front Pharmacol (2021) 12:724306. doi: 10.3389/fphar.2021.724306 34790117 PMC8591126

[96] Schmohl JU Vallera DA. CD133, selectively targeting the root of cancer. Toxins (Basel) (2016) 8(6):165. doi: 10.3390/toxins8060165 PMC4926132 27240402

[97] Wang Y Chen M Wu Z Tong C Dai H Guo Y. CD133-directed CAR T cells for advanced metastasis malignancies: A phase I trial. Oncoimmunology (2018) 7(7):e1440169. doi: 10.1080/2162402X.2018.1440169 29900044 PMC5993480

[98] Bai Y Kan S Zhou S Wang Y Xu J Cooke JP. Enhancement of the *in vivo* persistence and antitumor efficacy of CD19 chimeric antigen receptor T cells through the delivery of modified TERT mRNA. Cell Discov (2015) 1:15040. doi: 10.1038/celldisc.2015.40 27462436 PMC4860832

[99] Long AH Haso WM Shern JF Wanhainen KM Murgai M Ingaramo M. 4-1BB costimulation ameliorates T cell exhaustion induced by tonic signaling of chimeric antigen receptors. Nat Med (2015) 21(6):581–90. doi: 10.1038/nm.3838 PMC4458184 25939063

[100] Zhang ZZ Wang T Wang XF Zhang YQ Song SX Ma CQ. Improving the ability of CAR-T cells to hit solid tumors: Challenges and strategies. Pharmacol Res (2022) 175:106036. doi: 10.1016/j.phrs.2021.106036 34920118

[101] Vincent L Kermani P Young LM Cheng J Zhang F Shido K. Combretastatin A4 phosphate induces rapid regression of tumor neovessels and growth through interference with vascular endothelial-cadherin signaling. J Clin Invest (2005) 115(11):2992–3006. doi: 10.1172/JCI24586 16224539 PMC1253622

[102] Deng C Zhao J Zhou S Dong J Cao J Gao J. The vascular disrupting agent CA4P improves the antitumor efficacy of CAR-T cells in preclinical models of solid human tumors. Mol Ther (2020) 28(1):75–88. doi: 10.1016/j.ymthe.2019.10.010 31672285 PMC6953963

[103] Ferrara N Gerber HP LeCouter J. The biology of VEGF and its receptors. Nat Med (2003) 9(6):669–76. doi: 10.1038/nm0603-669 12778165

[104] Xing H Yang X Xu Y Tang K Tian Z Chen Z. Anti-tumor effects of vascular endothelial growth factor/vascular endothelial growth factor receptor binding domain-modified chimeric antigen receptor T cells. Cytotherapy (2021) 23(9):810–9. doi: 10.1016/j.jcyt.2021.05.008 34244079

[105] Kalluri R Zeisberg M. Fibroblasts in cancer. Nat Rev Cancer (2006) 6(5):392–401. doi: 10.1038/nrc1877 16572188

[106] Yang X Lin Y Shi Y Li B Liu W Yin W. FAP promotes immunosuppression by cancer-associated fibroblasts in the tumor microenvironment *via* STAT3-CCL2 signaling. Cancer Res (2016) 76(14):4124–35. doi: 10.1158/0008-5472.CAN-15-2973 27216177

[107] Ebert LM Yu W Gargett T Toubia J Kollis PM Tea MN. Endothelial, pericyte and tumor cell expression in glioblastoma identifies fibroblast activation protein (FAP) as an excellent target for immunotherapy. Clin Transl Immunol (2020) 9(10):e1191. doi: 10.1002/cti2.1191 PMC7557106 33082953

[108] Hiltbrunner S Britschgi C Schuberth P Bankel L Nguyen-Kim TDL Gulati P. Local delivery of CAR T cells targeting fibroblast activation protein is safe in patients with pleural mesothelioma: first report of FAPME, a phase I clinical trial. Ann Oncol (2021) 32(1):120–1. doi: 10.1016/j.annonc.2020.10.474 33098996

[109] Grasso CS Giannakis M Wells DK Hamada T Mu XJ Quist M. Genetic mechanisms of immune evasion in colorectal cancer. Cancer Discovery (2018) 8(6):730–49. doi: 10.1158/2159-8290.CD-17-1327 PMC5984687 29510987

[110] Katz SC Hardaway J Prince E Guha P Cunetta M Moody A. HITM-SIR: phase ib trial of intraarterial chimeric antigen receptor T-cell therapy and selective internal radiation therapy for CEA(+) liver metastases. Cancer Gene Ther (2020) 27(5):341–55. doi: 10.1038/s41417-019-0104-z 31155611

[111] Morris EC Neelapu SS Giavridis T Sadelain M. Cytokine release syndrome and associated neurotoxicity in cancer immunotherapy. Nat Rev Immunol (2022) 22(2):85–96. doi: 10.1038/s41577-021-00547-6 34002066 PMC8127450

[112] Brudno JN Kochenderfer JN. Recent advances in CAR T-cell toxicity: Mechanisms, manifestations and management. Blood Rev (2019) 34:45–55. doi: 10.1016/j.blre.2018.11.002 30528964 PMC6628697

